# Postscript to Invertebrate Welfare: “We Have Met the Enemy and He Is Us”

**DOI:** 10.3390/ani14142082

**Published:** 2024-07-17

**Authors:** William Winlow, Jennifer Mather, Anna Di Cosmo

**Affiliations:** 1 Department of Biology, University of Naples Federico II, 80126 Naples, Italy; 2 Institute of Ageing and Chronic Diseases, University of Liverpool, Liverpool L69 3BX, UK; 3 Department of Psychology, University of Lethbridge, Lethbridge, AB T1K 3M4, Canada

Through this collection of papers, we have considered in depth the effects that humans have on invertebrate welfare in a variety of contexts. For instance, we have discussed how these effects manifest in breeding or display facilities and in testing and research laboratories, as well as in the wild. The articles comprising this collection, though few, span species of different phyla and in conditions, yet they represent only a sample of contexts relevant to this research topic. To facilitate this discussion, we have grouped these scenarios into representative tables. For those who know little about invertebrates, References [1,2] provide ample background for a sufficient understanding.

## 1. How Humans View Invertebrates (See Table 1)

The field of animal welfare is an inclusive one, containing ideas from philosophy, ethics, law, agriculture and fisheries, tourism—including zoos and aquaria—and even veterinary care. World Animal Protection defines welfare as follows: “The physical and psychological well-being of an animal. It is good or high if the individual is fit, healthy, free to express natural behavior, free from suffering and in a state of wellbeing.” It is likely that the first clear definition of the term was that of the Brambell Committee in the UK, which investigated the welfare of agricultural animals, leading to the recognition of the often cited five freedoms [3]: freedom from (a) hunger and thirst, (b) discomfort, (c) pain, injury, and distress, (d) and fear, and (e) freedom to express normal behaviour. However, some have reported that these factors are too focused on negative freedom; Dawkins [4] suggested that domestic animals have a high degree of plasticity, and that we should evaluate not their ‘natural behaviour’ but what the animals themselves value as experiences. Mellor [5], supported by Miller and Chinnadurai [6], proposed that we focus instead on five provisions: these include good nutrition, a satisfactory environment, good health, appropriate behaviour, and positive mental experiences. In either case, what these two definitions share is the necessity to evaluate whether animals are doing well.

By virtue of living in the Anthropocene era [7], everything on and in the earth is strongly affected by human attitudes and actions. Tracking human attitudes is paramount, as the public view of ethics changes when new information is revealed, and human views necessarily influence scientific practice [8].

**Table 1:** Studies on how humans view invertebrates.

**•** Ethics and Invertebrates: The Problem Is Us, by **Jennifer A. Mather**
**•** The Long Road from Religious and Ethical Traditions to Welfare of Invertebrates, by **Jennifer A. Mather**

## 2. Attitudes to Captive Invertebrates (See Table 2)

Ethical concerns, public opinion, legal regulations, and material values all work together to ‘keep us in the dark’ about invertebrate welfare [9]. And yet the biggest problem we face with regard to this field of study is the lack of available information about invertebrates and their need for welfare consideration. What are we doing (or not doing) to them? In what ways are we influencing them? What do we need to do for them? 

**Table 2:** Research on attitudes toward captive invertebrates.

**•** Invisible Invertebrates: The Welfare of Invertebrates in Public Aquaria, by **Kerry Perkins**
**•** Assessing the Welfare of Captive Group-Housed Cockroaches, *Gromphadorhina oblongonota*, by **Danielle Free** and **Sarah Wolfensohn**
**•** Ethical Considerations for Echinoderms: New Initiatives in Welfare, by **Augusto César Crespi-Abril** and **Tamara Rubilar**

It is of course most important to consider the welfare of invertebrates when they are our captives and when we have direct control of them. A portion of this collection features situations involving captive invertebrates, including those on display and those used in science. Similar contexts that we were not able to discuss in this collection include other ecotourism practices, such as insectariums and butterfly farms [10]. A more extensive emerging concern is the agricultural use of insects, with already one trillion insects being used as human food [11]. 

## 3. Care of Captive Invertebrates (See Table 3)

The 3R principles of welfare—reuse, reduce, and replace—[12] are sometimes interpreted to mean “replace the important and sentient mammals in testing with unimportant invertebrates”. However, modern research advances in animal cognition have made it clear that complex information processing is not just the territory of vertebrates. Cephalopod molluscs and decapod crustaceans in particular have been shown to be much more intelligent and sensitive than we previously assumed [13]. 

**Table 3:** Studies on pain and anaesthesia.

**•** Behavioural Indicators of Pain and Suffering in Arthropods and Might Pain Bite Back? by **Robert W Elwood**
**•** The Use of Isoflurane and Adjunctive Magnesium Chloride Provides Fast, Effective Anaesthetization of Octopus vulgaris, by **Anna Di Cosmo**, **Valeria Maselli**, **Emanuela Cirillo**, **Mariangela Norcia**, **Heethaka K. S. de Zoysa**, **Gianluca Polese** and **William Winlow**
**•** Effects of acetic acid and morphine in shore crabs, *Carcinus maenas*: implications for the possibility of pain in decapods, by **Stuart Barr and Robert W. Elwood**

It is surely the responsibility of humans, and particularly of scientists, to take the welfare of invertebrates seriously and to treat them well in captivity and in the wild. Because of this, we ought to focus on both pain and awareness, and how we might measure and prevent them. Other papers have zeroed in on wider solutions; for example, Crook [14] focuses on the future responsibilities of young researchers and veterinary groups [15].

## 4. Human Influences on Wild Invertebrates (See Table 4)

Although our initial and continuing focus has been on the welfare of animals under direct human control, this has to expand. One direction of expansion has gone beyond the direct human influence and control of animals in captivity to the indirect influence we have on ‘wild’ animals across ecosystems globally. In the Anthropocene era [7], human influence is pervasive throughout all regions on Earth. This includes the inadvertent and planned modification of the physical environment and the unplanned effects that come with this, such as tourism, pollution, and climate change. These factors have significant real-life consequences, regardless of whether they were planned, and they matter to animals outside of our direct control.

**Table 4:** Papers on invertebrates in the wild.

**•** Effects of Artificial Light at Night on Fitness-Related Traits of Sea Urchin (*Heliocidaris crassispina*), by **Xiuwen Xu**, **Zexianghua Wang**, **Xiuqi Jin**, **Keying Ding**, **Jingwen Yang** and **Tianming Wang**
**•** Assessing Negative Welfare Measures for Wild Invertebrates: The Case for Octopuses, by **Michaella P. Andrade**, **Charles Morphy, D. Santos**, **Mizziara M. M. De Paiva**, **Sylvia L. S. Medeiros**, **C. E. O’Brien**, **Françoise D. Lima**, **Janaina F. Machado** and **Tatiana S. Leite**
**•** It Is Not Only Data—Freshwater Invertebrates Misused in Biological Monitoring, by **Paweł Koperski**
**•** Increasing Risks to the Health of the Invertebrates—Balancing between Harm and Benefit, by **Tatiana V. Kuznetsova**, **Valentina A. Kudryavtseva** and **Larisa L. Kapranova**

Because the scope of this research topic is so wide, we have been able to feature research from diverse backgrounds. These include accidental pollution, inadvertent mistreatment, and the careless use of chemicals and capture devices. It was also necessary to expand the scope of this collection to include the individual influences of light pollution [16] and chemical toxicity [17]. In addition, we have added references regarding their effects on invertebrates in whole land [18] and marine [19] ecosystems. Furthermore, we must not ignore the effects of temperature increase in this period of climate change, which produces hypoxic and acidic marine and freshwater environments, which are deleterious to aquatic life [20,21].

In our activity, we affect the whole planet [7], and invertebrates make up 98% of the animals on and in it. Of course this matters. In conclusion, “**We have met the enemy and he is us**” [22].
